# Yuanhuacin and Related Anti-Inflammatory and Anticancer Daphnane Diterpenes from Genkwa Flos—An Overview

**DOI:** 10.3390/biom12020192

**Published:** 2022-01-23

**Authors:** Christian Bailly

**Affiliations:** OncoWitan, Scientific Consulting Office, 59290 Lille, France; christian.bailly@oncowitan.com

**Keywords:** yuanhuacin, anti-inflammatory, anticancer, *Daphne genkwa*, sesquiterpene lactone

## Abstract

The dried flower buds of the plant *Daphne genkwa* Sieb. et Zucc. have been largely used in traditional Chinese medicine for the treatment of inflammatory diseases. Numerous diterpenoids have been isolated from the Genkwa Flos (yuanhua in Chinese), including a series of daphnane-type diterpene designated as yuanhuacin (YC, often improperly designated as yuanhuacine) and analogues with a patronymic name. The series includes ten daphnane-type diterpenes: yuanhuacin, yuanhuadin (YD), yuanhuafin (YF), yuanhuagin (YG), yuanhuahin (YH), yuanhuajin (YJ), yuanhualin (YL), yuanhuamin (YM), yuanhuapin (YP), and yuanhuatin (YT). They are distinct from the rare flavonoid yuanhuanin. The series comprises several anticancer agents, such as the lead compound YC, which has revealed potent activity in vitro and in vivo against models of lung and breast cancers. The main signaling pathways implicated in the antitumor effects have been delineated. Protein kinase C is a key factor of activity for YC, but in general the molecular targets at the origin of the activity of these compounds remain little defined. Promising anticancer effects have been reported with analogues YD and YT, whereas compounds YF and YP are considered more toxic. The pharmacological activity of each compound is presented, as well as the properties of Genkwa Flos extracts. The potential toxic effects associated with the use of these compounds are also underlined.

## 1. Introduction

**Figure 1:**
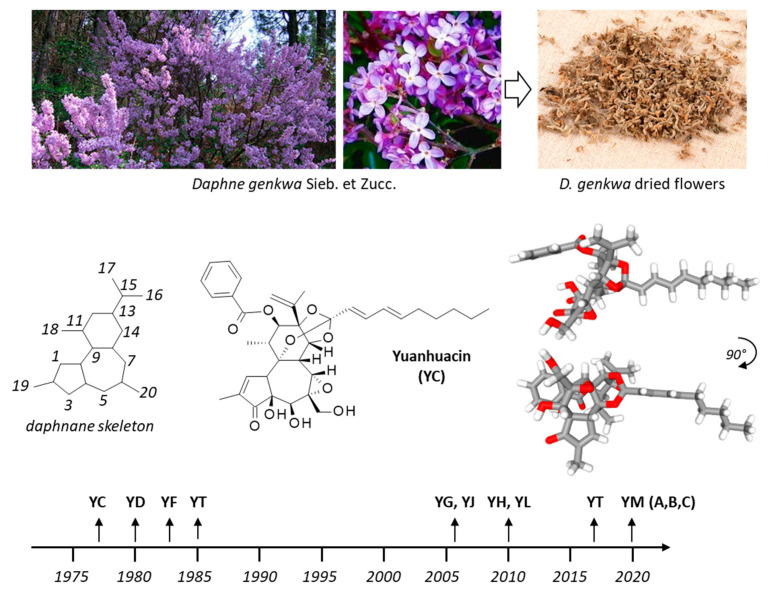
The flowering plant *Daphne genkwa* Sieb. et Zucc. (Thymelaeaceae) and the dried flowers from which the lead product yuanhuacin (YC, often designated yuanhuacine) is extracted. The chemical structure of YC is shown, together with a molecular model of the compound. YC was discovered in 1977, followed by the other yuanhua*X*in derivatives, until the recent discovery of yuanhuamin (YM) A, B and C in 2020. Yuanhuacin (YC), yuanhuadin (YD), yuanhuafin (YF), yuanhuagin (YG), yuanhuahin (YH), yuanhuajin (YJ), yuanhualin (YL), yuanhuamin (YM), yuanhuapin (YP), and yuanhuatin (YT).

The dried flower bud from the plant *Daphne genkwa* Sieb. Et Zucc. (Thymelaeaceae) is used as a traditional herbal medicine in Asian countries for the treatment of inflammation-related symptoms and diseases [1]. The plant can be found largely in the east part of China, notably in the provinces of Anhui, Fujian, Hubei, Jiangsu, Sichuan, Shandong, and Zhejiang. The buds are collected before blooming of the flowers and dried in the sun. The flower bud, known as Genkwa Flos (yuanhua in Chinese), is used to treat arthritis and in diuretic, antitussive, and expectorant preparations [2,3,4]. In addition, marked anticancer effects have been reported when using Genkwa Flos extracts [5,6]. The plant is also used in multiherbal preparations, such a Shizaotang (also known as Sibjotang) used to treat malignant pleural effusions. This herbal preparation is made from *Daphne genkwa* Sieb. et Zucc. and three other plants (*Euphorbia kansui* S.L.Liou ex S.B.Ho, *Euphorbia pekinensis* Rupr., and *Ziziphus jujuba* Mill.) [7,8]. However, Genkwa Flos has been reported to have significant hepato-nephrotoxicity [9]. To reduce the toxicity of the plant extract, a processing treatment with vinegar can be performed, without reducing the antitussive and expectorant actions [10,11]. The vinegar processing markedly changes the metabolite profile of the extracts, reducing notably the content of the daphnane diterpenes yuanhuacin (Figure 1) and genkwadaphnin which have been considered as toxic compounds [12,13]. The processing with vinegar enhances the bioavailability of the flavonoids genkwanin and 3’-hydroxygenkwanin but reduces the bioavailability of yuanhuacin and genkwadaphnin [14].

**Figure 2:**
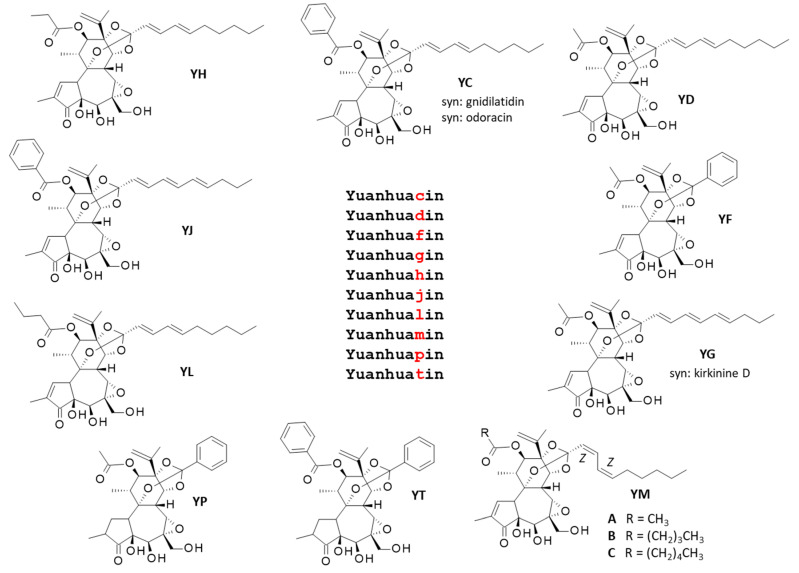
The yuanhua*X*in family (Y*X*) of compounds. All compounds are 6-epoxy-daphnanes.

Many bioactive compounds have been isolated from the roots of *Daphne genkwa* Sieb. et Zucc., notably a series of sesquiterpenoids with anti-inflammatory and cytotoxic properties. One can refer to (i) daphneaines A-to-G, and specifically daphneaine F which has revealed marked anti-inflammatory effects [15], (ii) genkwanoids A-K endowed with neuroprotective effects [16], and (iii) the guaiane-type sesquiterpenoids called daphne A-G, with cytotoxic and neuroprotective properties [17,18]. Daphnane-type diterpenoids are abundant and structurally diversified in *D. genkwa* extracts [19,20]. Here, we wanted to refer specifically to a family of little studied products structurally related to yuanhuacin (YC). There are ten related products which, in name only, differ from yuanhuacin only by a single letter, as indicated in Figure 2. They are daphnane-type diterpenes, such as yuanhuadin and yuanhuatin, also found in flower buds of *D. genkwa* [21,22] and they share a common 6-epoxy-daphnane skeleton. In sharp contrast, the product called yuanhuanin is a flavonoid, with a totally distinct structure. The similarities between the different chemical names do not facilitate the proper identification of the compounds, particularly as several of these compounds have synonym names as well. The present review clarifies the situation, to help the proper identification and comparison of the compounds. Their pharmacological properties have been analyzed.

## 2. Yuanhuacin, a Potent Anticancer Agent

The discovery of YC dates from 1977, when the compound was first isolated from the root of Yuan-Hua (*Daphne gemkwa*) by Chinese scientists from the Shanghai institute of materia medica [23], but the correct structure was refined much later [24]. Early studies refer to the anti-abortive effects of the plant yuanhua and the product YC [25,26]. In the 1970–1980s, yuanhua preparations such as elixirs of yuanhua flowers and yuanhua roots were largely used in China to induce mid-term abortion [27,28]. At that time, several antifertility compounds were isolated from the plant, including YC but also yuanhuadin (YD) and yuanhuafin (YF) (Figure 2). In the late 1980s, yuanhuacin-containing films have been developed for menstrual induction or to induce termination of early pregnancy [29,30]. They are no longer used today.

A remark about the nomenclature of these natural products must be pointed out here. In the literature, yuanhuacin is often improperly designated yuanhuacine (with a “*e*” at the end of the word). The same for the other compounds in the series. *Sensu stricto* the name of all these compounds should be written without a “*e*” at the end, because the terminology *-ine* is usually reserved for alkaloids. YC is not an alkaloid, and for this reason the compound should be named yuanhuacin. This terminology has been adopted here for all compounds in the series (Figure 2).

**Figure 3:**
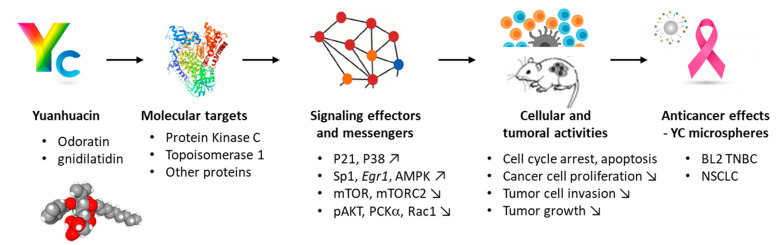
An illustration of the mechanism of anticancer action of yuanhuacin (YC), also known as odoratin or gnidilatidin. Binding of YC to protein kinase C, and possibly to the DNA-binding enzyme topoisomerase 1, triggers different cellular effects inducing upregulation of proteins such as p21, P38, and transcription factor Sp1, and downregulation of phosphor-AKT, PKCa and Rac1. These biochemical activities translate into cellular effects, as indicated, and lead to inhibition of tumor growth in xenografted mice model of cancer. YC has revealed prominent activities in basal-like 2 triple negative breast cancer (BL2 TNBC) and in specific models of non-small cell lung cancer (NSCLC).

The procedures to extract YC from the plant have been optimized [31] and analytical methods to evaluate the YC content of the plant have been refined [32]. YC can be easily obtained from the air-dried flowers of *Daphne genkwa*. In the original study, 9 kg of dried flowers afforded 82 mg of the pure compound (i.e., roughly 9 mg/kg of dried flowers). YC was initially characterized as an inhibitor of DNA synthesis and an antileukemic agent, moderately active in vivo in the P388 leukemia model [33,34]. Over the past twenty tears, the knowledge of the mechanism of action of the product has been refined. YC was identified as an antagonist of protein kinase C [35] and later as a modest inhibitor of the DNA-binding enzyme topoisomerase 1 [36]. Topoisomerase 1 is a major anticancer target, being notably the main target of camptothecin-based drugs such as topotecan and irinotecan used to treat cancer [37]. Therefore, it is conceivable that the anticancer properties of YC derive from topoisomerase 1 inhibition, at least in part. However, obviously, the mode of action of YC is multifactorial and multi-targeted (Figure 3).

The antiproliferative and anticancer activities of YC have been characterized using different cell and animal models (Table 1). Inhibition of cancer cell proliferation has been evidenced using P-388 and L-1210 murine leukemia cells [38] and A549 human lung cancer cells [39]. In the latter case, a highly potent inhibition of cell growth was evidenced in the presence of YC, with an IC_50_ values of 19 nM, whereas the compound presented no cytotoxic effect against MRC5 human normal lung epithelial cells [39]. However, heterogenous results have been reported. Li and coworkers showed that YC displays prominent antiproliferative against a variety of cancer cell lines, including colorectal (Colo 205 and HT29), breast (MCF7), melanoma (B16 and A2058), but not against A549 lung cancer cell surprisingly [6]. In the same vein, the antiproliferative efficacy of YC was found to be highly variable when using several non-small cell lung cancer (NSCLC) cell lines, with IC_50_ varying from 9 nM with H1993 cells (the most sensitive) to 16.5 μM with H358 cells (the less sensitive) and intermediate IC_50_s with other cell lines (A549, IC_50_ = 30 nM; H1299 and Calu-1 cells, IC_50_ = 4 μM) [40]. YC is not a non-specific cytotoxic agent; the cytotoxicity is extremely cell-type dependent, and in general, models of lung cancer respond well to YC. A robust anticancer activity has been observed when using the Lewis Lung carcinoma (LLC) model in mice with a reduction of tumor growth of 48% and 63% upon treatment (ip) with YC at 0.1 and 0.5 mg/kg, respectively [41].

A study performed with T24T bladder cancer cells (IC_50_ = 1.8 μM) and HCT116 colon cancer cells (IC_50_ = 14.3 μM) has confirmed the anti-proliferative potency of YC and the significant variability from one cell line to another [44]. With both T24T and HCT116 cells, the compound induced a G2/M cell cycle arrest coupled to an upregulation of the cyclin-dependent kinase inhibitor p21. A mechanism has been proposed whereby YC induces activation of p38 mitogen-activated protein kinase (MAPK), stabilization of transcription factor Sp1, and induction of p21 expression [44]. However, the mechanism of action of YC remains superficially known at present and no firm molecular target has been described. The inhibitory activity against topoisomerase 1 is too weak to explain the high potency of the compound. There must be other molecular targets. The compound acts on multiple signaling pathways, depending on the cancer cell type considered and the cell growth conditions. For example, it has been shown that YC can activate AMP-activated protein kinase (AMPK) and suppresses the mTORC2 (mammalian target of rapamycin complex-2)-mediated downstream signaling pathway in H1993 human non-small cell lung cancer (NSCLC) cells. In this case, YC modulated the expression of several major proteins implicated in cell proliferation and in cell mobility, such as deactivation of mTOR (mammalian target of rapamycin) and decreased expressions of p-Akt, PKCα and phosphor-PKCα (protein kinase C alpha), and Rac1 (p-ras-related C3 botulinum toxin substrate 1). This latter protein is a major contributor of cancer cell invasion and dissemination. The downregulation of Rac1 by YC can be linked to the impact of the compound on modulation of the actin cytoskeleton and the antimetastatic effect. Low concentrations of YC, in the 10–40 nM range, were sufficient to reduce considerably the migration and invasion of H1993 cells in vitro. This effect is interesting because it led to synergistic combinations of YC and the kinase inhibitor gefitinib in this NSCLC cellular model [40].

**Figure 4:**
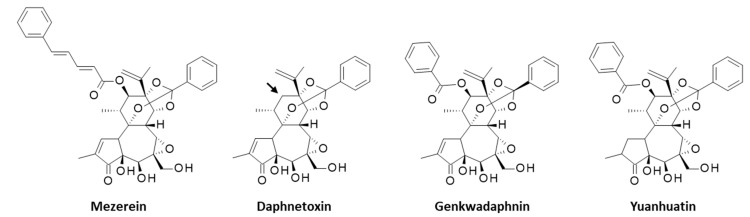
Structures of three sesquiterpene lactone structurally related to YT. The compounds grouped as yuanhua*X*in family, including yuanhuatin (YT), only differ from daphnetoxin by oxygenation at the C-12 position (black arrow). In this sense, we could consider that mezerein and genkwadaphin also belong to the yuanhua*X*in family, as they bear a -OR substituent at C-12.

Year after year, evidence accumulate to demonstrate the potent antiproliferative effects of YC in a variety of cellular models and its activity in selected models of tumors in mice (Table 1). The most recent study refers to the selective inhibition of BL2-subtype (basal-like 2) triple negative breast cancer (TNBC) coupled to an induced differentiation of THP-1 monocytes into adherent myeloid cells [43]. This work is remarkable, highlighting the major potency of YC at inhibiting proliferation of BL2-type TNBC cells, such as HCC1806 cells (IC_50_ = 1.6 nM), compared with other non-BL2 TNBC cells, such as mesenchymal-subtype MDA-MB-231 cells (IC_50_ > 3 μM). The BL2 subtype selectivity has been associated with the induction of protein kinase C (PKC). The pre-treatment of HCC1806 cells with a large-spectrum PKC inhibitor reduced considerably (by 80%) the antiproliferative activity of YC, thus suggesting that PKC inhibition is a prime element of the drug action (Figure 3). These effects translated into a very significant antitumor activity of YC in vivo, in mice xenografted with HCC1806 tumor cells. The treatment of mice with YC (intraperitoneal injection at 0.7–1 mg/kg) reduced tumor growth more efficiently than the reference drug paclitaxel. However, the compound was found to be quite toxic to mice, with significant weight loss and occasionally the death of one mouse at 1 mg/kg [43]. The highly potent activity of YC against this model of BL2-subtype TNBC is promising and calls for further studies to appreciate the benefit/rick ratio of the natural product. The functioning of YC as a potential PKC agonist is entirely conceivable because there are closely related products known to function as PKC activator, such as mezerein (Figure 4) and daphnetoxin (isolated from the stem bark of *Daphne gnidium*) each acting against different PKC subtypes [45]. The PKC isoform-selectivity of YC warrants specific studies.

YC has been also isolated from the plant *Daphne odora* Thunb. and the product was named odoracin [46,47]. Similarly, YC has been isolated from different *Gnidia* species, such as G. *latifolia*, *G. glaucus* Fres, and *G. kraussiana*, and in this case the product was named gnidilatidin [48,49,50]. Therefore, the three names—yuanhuacin (frequently written yuanhuacine, with a “e” at the end of the word), odoracin, and gnidilatidin—are synonyms (Figure 2).

The name odoracin is rarely used and should not be confused with odoratin, which is a common name for plant natural products. The name odoratin is confusing because there are no fewer than six different compounds called odoratin, issued from different plants [51,52]. Parenthetically, there is another daphnane diterpenoid with the same molecular formula as odoracin and called odoratrin (both C_37_H_44_O_10_), also isolated from *Daphne odora* [47,53]. The name gnidilatidin has been used occasionally in a few publications. In particular, it was shown that gnidilatidin (YC) could inhibit the expression of the two metastasis-associated genes *Id2* and *Sytl2*, while upregulating the tumor suppressor gene *Egr1*. This daphnane diterpene has been identified also in the leaves of the Mediterranean medicinal plant *Thymelaea hirsuta* and found to inhibit melanogenesis in murine B16 melanoma cells [54]. More recently, it was found to contribute to the decrease in lung tumor formation in mice injected with B16 murine melanoma cells [55].

All the above information attests that YC displays marked cytotoxic activities in vitro and antitumor activities in vivo. The compound can efficiently reduce the growth of certain tumors in mice. However, the systemic administration of YC is generally accompanied with marked toxicities toward several organs, notably the liver, kidneys, and the reproductive system. Genkwa Flos is known to induce dysfunction of intestines and a global oxidative stress [56]. The various yuanhua diterpenoids have been incriminated in the in vivo toxicity; notably, compounds such as yuanhuafin (YF) and yuanhuapin (YP) both present at a much higher levels in blood compared with YC after administration of a total diterpenoid extract of Genkwa Flos to mice [57]. YC appears to be less toxic than YF and YP, but the intestinal absorption of the compound is very poor, as it is the case also for yuanhuadin (YD) and yuanhuatin (YT) [57]. Moreover, a vinegar processing of *Daphne genkwa* extracts has been shown to further reduce the bioavailability of YC [14]. The T_1/2_ measured after an intravenous administration of YC in rats is relatively short (5.3 h), but the pharmacokinetic parameters can be significantly improved when using an inhaled powder sample of YC for the pulmonary administration, to reach a T_1/2_ of 63.9 h. The formulation for pulmonary administration, obtained upon mixing of YC with lactose [58], provides a better exposure to the compound, increasing the T_1/2_ but reducing significantly the C_max_ (from 185.3 to 24.9 ng/mL for the *iv* and pulmonary administration, respectively), thereby reducing potentially the risk of toxicity by avoiding high blood concentrations [59]. There is also the possibility to use specific microspheres loaded with YC to deliver the compound to the lung. Small-sized microspheres (with an average diameter of 9.0 μm) made from PLGA (poly (d,l-lactide-co-glycolide)) have been developed for this purpose. They offer an efficient delivery of the compound to the lung [60]. PLGA-based nanocarriers and multifunctional nanoparticles can very significantly improve the delivery of inhaled YC to the lung [61].

## 3. YC and Analogues. The yuanhua*X*in Family (Y*X*)

Apart from YC (discussed above) and the flavonoid yuanhuanin (YN, discussed below), there are 9 naturally occurring derivatives with a closely related name: yuanhuadin (YD), yuanhuafin (YF), yuanhuagin (YG), yuanhuahin (YH), yuanhuajin (YJ), yuanhualin (YL), yuanhuamin (YM), yuanhuapin (YP), yuanhuatin (YT) (Figure 2). We can refer collectively to yuanhua*X*in to mention the complete family. Several of these compounds were first isolated in China from the roots and/or the flower buds of plant Yuan-Hua (*Daphne genkwa*) in the early 1980s and were characterized as antifertile diterpenes. This is the case for YD [62,63], YF [64,65], YT [66], and YP [67]. We will refer to each compound successively.

**Figure 5:**
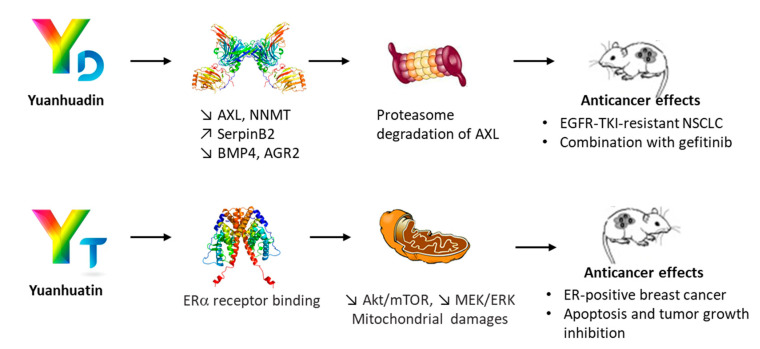
Illustration of the mechanism of anticancer action of yuanhuadin (YD) and yuanhuatin (YT). YD induces a downregulation of the AXL tyrosine kinase receptor (often overexpressed in NSCLC cells) and the enzyme nicotinamide N-methyltransferase (NNMT). A concomitant upregulation of serine protease inhibitor SerpinB2 has been observed, together with a reduced expression of the bone morphogenetic protein 4 (BMP4) and anterior gradient protein 2 (AGR2). The compound triggers the proteasomal degradation of AXL and the signaling effects lead to a significant activity of YD in models of NSCLC resistant to the EGFR inhibitor gefitinib. The combination of YD and gefitinib is synergistic. YT has been reported to bind to the estrogen receptor-alpha (ERα) to trigger mitochondrial dysfunctions in ERα^+^ breast cancer cells.

Yuanhuadin (YD) was first described in 1980, isolated from the roots of *Daphne genkwa* and considered, together with YC, to induce abortion [62,68]. YC is the major Daphne diterpene ester in the roots of the plant whereas YD is the major component in the flower buds [69]. YD was found to be a little more cytotoxic to cancer cells (A549) than YC but also more toxic toward non-cancer MRC-5 cells [39]. Nevertheless, the compound has been shown to exert significant antitumor effect in vivo, reducing the growth of human A549 lung tumor in mice upon oral administration (0.5 mg/kg). YD was found to induce cell-cycle arrest and to suppress the Akt/mTOR and EGFR signaling pathways. A synergistic effect was observed upon combination with the epidermal growth factor receptor (EGFR) tyrosine kinase inhibitor gefitinib [70]. In addition, YD was found to be active against gefitinib-resistant non-small cell lung cancer (NSCLC) in mice, via the targeting of the AXL receptor tyrosine kinase, frequently overexpressed in NSCLC cells [71,72]. YD induced a downregulation of AXL and concomitant upregulation of SerpinB2, promoting the proteolytic degradation of the AXL receptor and accelerating the receptor turnover (Figure 5). It could be an option to improve treatment of EGFR-TKI-resistant NSCLC [73,74]. The anticancer activity of YD toward EGFR-TKI-resistant cells and tumors is particularly interesting and has boosted a number of studies to define the molecular factors implicated in the activity of the compound. Lee and coworkers have identified several key factors playing a role in the restoration of EGFR-TKIs sensitivity, such as (i) the metabolic enzyme nicotinamide N-methyltransferase (NNMT) which expression is suppressed by YD in EGFR-TKI-resistant NSCLC cells [75], (ii) the bone morphogenetic protein BMP4 which expression is also drastically reduced in the same cells upon treatment with YD [76] and recently (iii) the anterior gradient protein 2 (AGR2) which is considered a marker of tumor aggressiveness, downregulated by YD in NSCLC-resistant cells [77]. YD also displays anti-inflammatory effects, reducing the production of nitric oxide (NO) in LPS-activated microglial BV2 cells with an efficacy comparable to that of YT [21]. In another study, YD showed an anti-inflammatory potential lower than that of the analogues YC and YG [78].

Yuanhuafin (YF) was described first in 1982 [64], but the compound has received very little attention thus far. It was re-isolated in 2010 from the flower buds of *Daphne genkwa* together with the analogues yuanhuapin (YP) and yuanhuatin (YT) [79]. YF and YP are both considered to be toxic compounds, present at a low level, but more toxic than the other diterpenoids in the plant [57]. After oral administration to rats, YP is slowly absorbed but it is also slowly eliminated and biotransformed. Up to 14 metabolites have been identified, resulting from hydroxylation, methylation, glucuronidation, and cysteine conjugation reactions [80,81]. This compound is probably the most toxic in the Y*X* series, being particularly harmful to the liver and the digestive tract, due to YP-induced modifications of the metabolism of amino acids, lipids, and carbohydrates. Perturbations of the gut microflora have been also mentioned with YP [81]. It has been reported that this compound is a highly potent ligand for protein kinase C (PKC, K_i_ = 0.48 nM) and an efficient cytotoxic agent against cancer cells (EC_50_ = 7 nM against K562 leukemia cells) [82]. The synthesis of YP and analogues has been achieved, representing a real tour de force. The chemical approach proposed enables the rational design of analogues maintaining a high potency against PKC but less toxic [82,83]. This very lipophilic compound (log P = 4.32) may have drug safety issues [84]; more hydrophilic analogues would be probably safer.

Yuanhuagin (YG) and yuanhuajin (YJ) were identified together with YC, YD, YP, from *D. genkwa* [36,69]. YG is also known as kirkinine D (C_32_H_40_O_10_), a daphnane orthoester isolated from *Pimelea elongata* [85]. The antiproliferative activity of YG against different lung cancer cell lines proved to be significantly lower than that of its analogues YH and YL (both issued from *D. genkwa* [39]). For example, IC_50_ of 9, 4.7 and 0.3 μM were calculated with YH, YL, and YG, respectively, when using human H358 lung cancer cells [86]. Other cell lines were found to be more sensitive to YG than H358 cells, but in most cases, the compound was less cytotoxic than YH or YL. The latter compound, YL, is particularly interesting because it has shown marked synergistic effects when combined with established anticancer drugs such as gemcitabine and gefitinib [86].

Three compounds designated yuanhuamins A, B and C have been isolated recently from *D. genkwa* and a preliminary assessment of their anti-HIV activity has been reported. All three compounds seem to be extremely efficient at inhibiting HIV entry into cells, with IC_50_ < 1 nM [87]. However, no mechanism has been proposed for these compounds. Finally, we can refer to yuanhuatin (YT), first isolated from the flower buds of *D. genkwa* in 1985 [66] and further characterized from the stem and root portions of the plant in 2017, together with YC, YD and a few other anti-inflammatory diterpenes. The capacity of YT to reduce the production of proinflammatory cytokines was inferior to that of YD [21]. Nevertheless, YT revealed interesting anti-cancer properties, with a capacity to reduce the proliferation of estrogen receptor (ER)-positive MCF-7 breast cancer cells which were much more pronounced compared with the reference anticancer drug tamoxifen (IC_50_ = 0.62 and 14.43 μM for YT and tamoxifen, respectively (Figure 5). The compound induced mitochondria dysfunctions in cells, and a molecular modeling analysis has suggested that YT could bind to the ERα receptor, inducing a downregulation of the receptor and causing apoptotic cell death [22]. This compound, lacking a long alkyl side chain such as that possessed by most Y*X* derivatives (e.g., YC, YD, YG, YJ, YL) but bearing a phenyl group at position 1′, bears a structural analogy with YF and also with the related natural product genkwadaphnin (Figure 4), which is a remarkable anticancer agent [88,89]. Without a doubt, YT deserves further investigations has an anticancer agent. It is interesting to note that all compounds in the yuanhua*X*in family, and including also mezerein and genkwadaphnin, have the isopropyl group at C-13, and consequently violate the isoprene rule.

Different molecular effectors and signaling pathways have been implicated in the mechanism of action of YC, TD and YT (Figure 3 and Figure 5). YC would target primarily PKC whereas YT seems to affect essentially ERα. However, this does not necessarily mean that they have very different primary targets. For example, there is a crosstalk between PKC and ERα [90,91]. All the compounds may have the same targets, but some may show a higher affinity for one target than the others. In particular, the different individual PKC subtypes could play distinct roles in signal transduction activated by these compounds.

## 4. The Flavonoid Yuanhuanin (YN)

**Figure 6:**
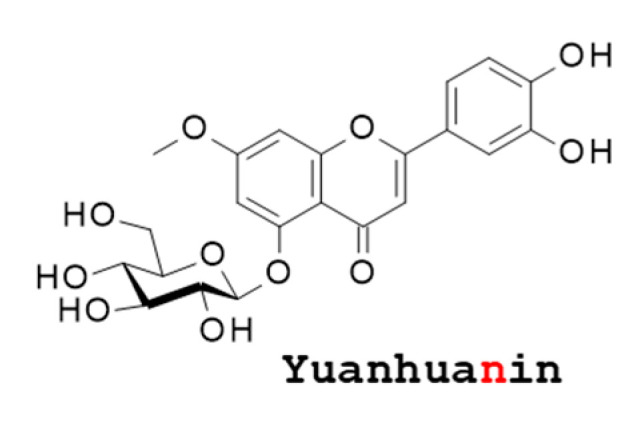
Structure of the flavonoid Yuanhuanin (YN). The name is reminiscent to the daphnane diterpenes shown in Figure 2, but this compound is a flavonoid.

YN (yuanhuanin, without a “e” at the end) is a glycosylated flavonoid corresponding to luteolin 7-methyl ether 5-*O*-β-d-glucoside (Figure 6). The origin of YN is uncertain. It was probably first isolated from the aerial parts of *Daphne genkwa* in China [92,93,94] and then from *D. sericea* [95]. The compound has been found also in the aerial parts of *D. papyracea* [96] and *D. pedunculata* H.F.Zhou ex C.Y.Chang [97,98]. In a recent study, the compound was found in the aerial parts of *D. sericea* Vahl collected in Italy [99]. The pool of flavonoids contained in *Daphne genkwa* displays marked anticancer properties and YN likely contributes to the anticancer and antimetastatic effects. However, there are other more potent flavonoids in the plant, such as the daphnodorins A–C [100]. Occasionally, the compound is referred to as luteolin 7-methyl ether 5-O-β-d-glucopyranoside (corresponding to YN), which can be found in the aerial parts of the plants *Diplomorpha canescens* [101] and *Coleus parvifolius* Benth. [102]. It has revealed a very weak antiviral activity against the integrase of the HIV-1 virus (IC_50_ = 70 μM). A molecular modeling analysis has suggested that YN could form stable complexes with the human epidermal growth factor receptor 2 (HER2) [103], but an experimental validation of this in silico proposal is awaited. YN remains a rare compound, not frequently studied and not particularly potent.

## 5. Conclusions

The present analysis clarifies the situation as regard the identification of the various yuanhuacin-like daphnane diterpenes isolated from the plant *Daphne genkwa*. The series of 11 compounds with a closely related name is unique, and somewhat amusing from the nomenclature point of view, but it creates confusion among members of the group. This peculiarity does not always facilitate the proper identification of the molecules and their distinction one from the other. The series includes 10 daphnane diterpenes and one outlier, the flavonoid yuanhuanin (YN).

In the daphnane series, the lead compound YC, also known as gnidilatidin, is a potent anti-inflammatory and anticancer agent. Robust proofs of the efficacy of the compound in several in vivo models of cancer have been accumulated (Table 1). The compound could be of interest for the treatment of specific lung and breast cancers. In particular, the high potency of YC to suppress the growth of BL2-subtype triple negative breast cancer (TNBC) is encouraging and promising [43]. However, at the same time, it is clear also that the compound can be toxic to non-cancerous cells and can cause side effects in animal models. The toxicity is even more pronounced with the analogues YP and YF [57]. The unwanted toxicity of YC can be reduced when using microspheres and/or functionalized nanoparticles to better control the delivery of the compound, notably to the lung system [61]. Nevertheless, these compounds remain difficult to handle, probably not well adapted as single molecular drug entities for the treatment of cancer. However, they can provide ideas for drug design. These molecules are structurally complex, not easily accessible from a chemical point of view, but the synthesis of analogues is feasible [82].

The series can be divided into two groups. On the one hand, the derivatives bearing a long unsaturated alkyl side chain at position 1′ (YC, YD, YG, YH, YJ, YL, YM) which could facilitate their anchorage to biological membranes and the encapsulation into lipid vesicles. These compounds are almost totally insoluble in aqueous media. On the other hand, the derivatives with a phenyl group at the 1′ position (YF, YP, YT) which are a little more soluble in water (aqueous solubility: 2.42 vs. 0.02 mg/mL for YP and YJ, respectively [84]), but also toxic, notably for compounds YF and YP [57]. The toxicity of some of these compounds likely explain why the use of the medicinal plant *Daphne genkwa* is sometimes restricted. In particular, the herb yuanhua cannot be combined with Glycyrrhizae radix (licorice), another common traditional Chinese herbal medicine. The licorice-yuanhua herbal pair is incompatible due to major gastro-intestinal side effects [104,105]. The combination of these two plants causes colonic H_2_S metabolism disturbance [106]. *Daphne genkwa* can be combined with other plants, notably with *Euphorbia* species, but in most cases, the plant is used as a standalone medicine.

*Daphne genkwa* is formidable source of daphnane-type diterpenes and flavonoids [107]. Here, we focused on yuanhuacin and related compounds, but there are other daphnanes in the flower buds of *Daphne genkwa*, such as the genkwanines [74], daphneresiniferins A and B [42], acutilonine F, wikstroemia factor M [21], daphgenkins A–G [108], genkwadanes A–D [5], neogenkwanine I [109], genkwalathins A–B [110] and others [20]. There are also a few iso-derivatives, such as iso-YC and iso-YD and a compound designated yuanhuaphnin with anti-inflammatory properties (but it is mentioned in a single publication) [78]. There are also numerous neuro-protective guaiane-type sesquiterpenoids [16,17,18], and flavonoids potentially useful to combat inflammatory diseases, such as arthritis [4].

In brief, an overview of the yuanhua*X*in-type compounds isolated from Genkwa Flos (yuanhua) is provided, highlighting the anti-inflammatory and anticancer properties of the lead compound yuanhuacin (YC) and its congeners. Robust evidence of cytotoxic activities in vitro and anticancer activities in vivo has been gained for YC, YD, and YT notably, but these compounds should be used with care to control their potential toxic effects. A better knowledge of the molecular targets of YC and derivatives would be extremely useful to guide the pharmacological use of these natural products.

## Figures and Tables

**Table 1:** Antiproliferative and anticancer activities reported with YC in different models.

Cellular or Tumor Models	Observed Effects	References
Melanoma cells	YC and YD inhibit melanogenesis and B16 melanoma cell proliferation in vitro.	[42]
Breast cancer model (BC)	BC growth inhibition in vivo (MCF7 model) with a Genkwa Flos extract and characterization of YC	[6]
Triple-negative breast cancer cell (TNBC), basal-like 2 subtype	Potent activity of YC against the BL2 subtype of TNBC (nM IC_50_ against HCC1806 and HCC70 cell lines).	[43]
Human non-small cell lung cancer (NSCLC)	YC inhibits cell growth and actin cytoskeleton organization and reduces tumor growth in vivo (H1993 model).	[40]
	Comparison of YC, YD, YG, YL as inhibitors of A549 cell proliferation.	[39]
	YC inhibits Lewis lung carcinoma (LLC) growth in mice.	[41]
Colon cancer cells	Inhibition of HCT116 cell proliferation and G2/M cell cycle arrest induced by YC, via upregulation of p21 and downregulation of Sp1.	[44]
Promyelocytic leukemia	YC inhibits proliferation of P-388 and l-1210 murine leukemia cells in vitro.	[38]
	YC inhibits HL-60 cell growth and induces apoptosis.	[41]
